# East Line Medical Society

**Published:** 1888-07

**Authors:** 

**Affiliations:** Mt. Pleasant, Texas


### EAST LINE MEDICAL SOCIETY.

MT. PLEASANT, TEXAS, June 10, 1888.

The East Line Medical Association of Texas met at Mt. Pleasant on the 5th of June, and had a splendid meeting. About thirty members were present. Eleven new applications were received for membership. Some very valuable papers were read. Dr. Blythe, of Mt. Pleasant, read a most excellent paper on "Antiseptics," which showed close reading and hard study.

Dr. S. T. Musick, of Pittsburg, reported a case of ovariotomy.

Dr. Yoakum read a paper on "Foreign Bodies in the Nose," and reported four cases of the same.

Dr. C. O. Mathews read a valuable paper on "Hydrogen Gas as a Diagnostic Means in Intestinal Perforations," quoting extensively from Prof. Senn.

Dr. Westbrook quoted Prof. Gaston, of Atlanta, who gave a saline injection as diagnostic means.

Dr. Cunningham asked if it was right to probe a gun-shot wound?

Dr. J. T. Music answered, that we are not justified in probing gun-shot wounds.

Dr. Crutcher verbally reported a case of amputation of the upper third of humerus.

Dr. J. T. Musick verbally reported a case of tetanus from gun-shot wound, and asked the best known remedies.

Dr. M. Smith answered, that Prof. Hodgens of St. Louis, claims as a specific four drops of Fowler's solution of arsenic every four hours. Dr. J. T. Musick believes amputation to be the remedy.

Dr. J. T. Music reported, verbally, a case of lacerated perineum, including the sphincter ani and two inches of the rectum, four or five months after confinement. Operated; recovery.

Dr. J. R. Riddle reported a case of placenta prævia, "four months enciente," twice in same patient, resulting in death the last time by forcibly taking contents from uterus.

Dr. S. O. Moore verbally reported a case of a man who, while cutting wood, let the ax glance and cut off part of the moleolus. Excessive pains; had trouble with the chloroform the second time it was given. Believed it was caused by a shattered nervous system.

Dr. Yoakum reported two cases of constipation relieved by rectal injections of pure glycerine, reading an extract from the *Therapeutic Gazette* on the "Practical Application of Glycerine in Medicine," which brought out quite a discussion.

Dr. J. T. Musick agreed with the paper. Dr. M. Smith had tried it in several cases. In some it acted well; but in others, where he looked for good results, was sadly disappointed. He did not think so well of it. Dr. O. S. Moore had used it very successfully, by the stomach. He thought it a splendid remedy, especially in children. His best formula was:

| | | |
|---|---|---|
| ℞ | Creosote | gtt. ¼ |
| | Bismuth | gr. i. |
| | Glycerine | ʒ i. |

for a child 1 to 1½ years old.

Dr. Yoakum verbally reported a case in which a man was bitten on glans penis by a small tick, which had caused sloughing off of two-thirds of penis and nearly all of the scrotum, leaving only about an inch of integument on the upper side, next to the body; testicles hanging far below, impossible to cover them with flap. Query: What is best to be done in this case, castrate, or try plastic operation?

This closed the Section work. This Association is composed principally of young men, who are making long strides in advanced medicine. Many of them are going annually to the post-graduate schools of medicine. We meet at Greenville, Texas, and all the M. Ds. are urged to attend with us, on the first Tuesday in October, next, and give aid to those who are striving to lift our noble profession out of the reach of quacks and charlatans. "Come all."

A MEMBER.

